# Titration of SF3B1 Activity Reveals Distinct Effects on the Transcriptome and Cell Physiology

**DOI:** 10.3390/ijms21249641

**Published:** 2020-12-17

**Authors:** Karen S. Kim Guisbert, Isiah Mossiah, Eric Guisbert

**Affiliations:** Department of Biomedical and Chemical Engineering and Sciences, Florida Institute of Technology, Melbourne, FL 32937, USA; imossiah2016@my.fit.edu (I.M.); eguisbert@fit.edu (E.G.)

**Keywords:** SF3B1, heat shock response, HSR, alternative splicing, pladienolide B, nonsense-mediated decay, NMD, early transcription termination, proteostasis

## Abstract

SF3B1 is a core component of the U2 spliceosome that is frequently mutated in cancer. We have previously shown that titrating the activity of SF3B1, using the inhibitor pladienolide B (PB), affects distinct steps of the heat shock response (HSR). Here, we identify other genes that are sensitive to different levels of SF3B1 (5 vs. 100 nM PB) using RNA sequencing. Significant changes to mRNA splicing were identified at both low PB and high PB concentrations. Changes in expression were also identified in the absence of alternative splicing, suggesting that SF3B1 influences other gene expression pathways. Surprisingly, gene expression changes identified in low PB are not predictive of changes in high PB. Specific pathways were identified with differential sensitivity to PB concentration, including nonsense-mediated decay and protein-folding homeostasis, both of which were validated using independent reporter constructs. Strikingly, cells exposed to low PB displayed enhanced protein-folding capacity relative to untreated cells. These data reveal that the transcriptome is exquisitely sensitive to SF3B1 and suggests that the activity of SF3B1 is finely regulated to coordinate mRNA splicing, gene expression and cellular physiology.

## 1. Introduction

The generation of mature mRNA transcripts by spliceosome-mediated RNA splicing is a eukaryotic innovation in gene expression. Regulation of splicing by a suite of splicing factors enables the production of multiple mRNA transcripts from each gene through alternative splicing (reviewed in [1]). In humans, more than 95% of multiexon genes can be alternatively spliced, generating more than 100,000 alternative splicing events [2,3]. Regulation of alternative splicing serves as a central control point in gene expression whose effects can ripple out to affect every aspect of cell physiology, including development, tissue specificity, signaling and stress responses.

Alternative splicing can generate additional functional isoforms or nonfunctional transcripts destined for degradation by quality control pathways. Chief among these pathways is the nonsense-mediated decay (NMD) pathway that detects premature termination codons in the pioneer round of translation and then targets these messages for degradation (reviewed in [4,5,6,7]). Inappropriate messages that bypass mRNA quality control can generate proteins that cannot be folded properly. These aberrant proteins are then recognized and targeted for degradation by protein quality control pathways (reviewed in [8]). The splicing, RNA quality control and protein quality control pathways work coordinately to control expression. Therefore, the choice of splice site can profoundly affect the fate of a transcript and, thus, the fate of the cell.

Dysregulation of alternative splicing and mutations in splicing factors are associated with cancer (reviewed in [9,10]). Prominent among these splicing factors is SF3B1, which is commonly mutated in myelodysplastic syndrome with refractory anemia and ring sideroblasts (MDS-RARS), where the frequency of mutations is approximately 80% (reviewed in [11,12]). SF3B1 is a component of the U2 snRNP subunit of the spliceosome that is involved in recognition of the branchpoint sequence. Knockdown of SF3B1 reveals that SF3B1 regulates alternative splicing [13]. The cancer-associated mutations in SF3B1 also induce widespread changes to alternative splicing [14,15,16,17,18,19,20,21,22]. A large study examining mutations across 21 tumor types from nearly 5000 patient samples identified SF3B1 mutations across multiple cancer types (pan-cancer cohort), indicating that it is a frequent target of dysregulation in cancer progression [23]. Other factors also known to influence the same step in splicing as SF3B1, namely U2AF1 and SRSF2, are also frequently mutated in cancer [12,24]. However, mutations in these factors are never found within the same tumor sample, indicating the importance of this step in splicing and an intolerance for multiple defects at the same step. The most common SF3B1 mutations appear to be gain-of-function mutations [25]. Therefore, studies of these mutants have revealed important information about the role of SF3B1 in cancer but cannot reveal the role of SF3B1 in normal cellular physiology.

SF3B1 has also been identified as the target of a handful of small molecules with anticancer activity, including the macrolide pladienolide B (PB) (reviewed in [25,26]). PB was originally identified as a natural product with anticancer activity from *Streptomyces platensis* [27]. Mutations in *SF3B1* cause resistance to PB in WiDr and DLD1 colorectal cancer cell lines, indicating that SF3B1 is the primary target of PB [28]. Binding of PB to SF3B1 prevents it from accessing a critical conformation needed for its function [29,30]. 

The effects of SF3B1 inhibition have been interrogated prior to this work and have illuminated valuable information concerning the substrates and mechanisms of SF3B1 [13,14,15,16,17,18,19,20,21,22,31,32,33,34,35,36,37,38]. However, these studies were performed at high SF3B1 inhibitor concentrations or in cell lines where these inhibitors cause cytotoxicity. Notably, exposure of cancer cell lines to PB (typically 100 nM–1 μM) arrests the cell cycle and induces apoptosis [31,39]. Here, we add another dimension to the study of SF3B1 by analyzing the effects of low concentrations of PB in a cell line that is not derived from malignant tissue. 

We have previously shown that SF3B1 regulates a cytoprotective pathway known as the heat shock response (HSR) [40]. The HSR is a well-conserved pathway that is involved in maintaining protein-folding homeostasis, termed proteostasis [40,41]. Heat stress induces protein misfolding, which triggers the activation of the HSF1 transcription factor in a highly regulated process. We found that SF3B1 affects HSF1 through two distinct mechanisms: one involving regulation of HSF1 activity and the other involving regulation of HSF1 levels. Interestingly, the two different mechanisms display differential sensitivity to SF3B1 inhibitors, such that mild inhibition (1–10 nM) of SF3B1 only affects HSF1 activity but strong inhibition of SF3B1 (100 nM PB or siRNA knockdown) affects both HSF1 activity and HSF1 concentration. We reasoned that the HSR was not the only pathway sensitive to the degree of SF3B1 activity. Here, we identify other pathways that have differential sensitivity to SF3B1 using genomic techniques.

## 2. Results

### 2.1. Pladienolide B Affects Splicing but not Cell Viability in HEK293T Cells

**Figure 1:**
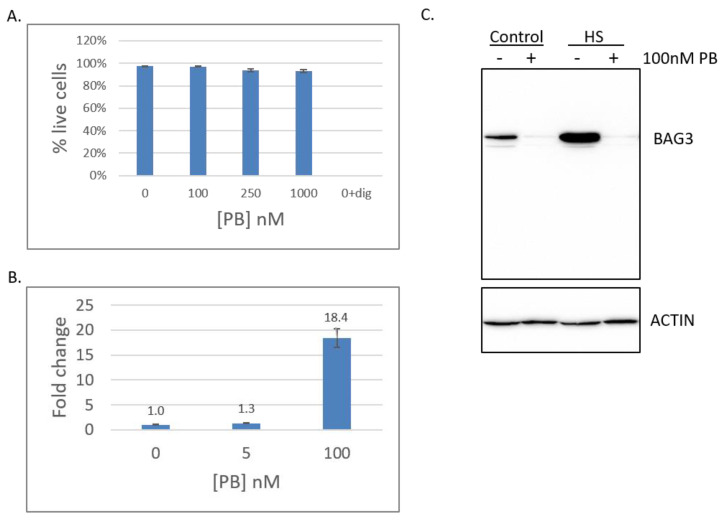
Pladienolide B (PB) inhibits SF3B1 without inducing cytotoxicity in HEK293T cells. (**A**) Cell viability after exposure for 16 h to PB using the Trypan Blue exclusion assay. Dig = digitonin control. (**B**) RT-qPCR quantitation of the RBM5 exon 5/7 alternatively spliced isoform after exposure to 5 or 100 nM PB. Fold change is shown relative to DMSO control cells. Error bars indicate SEM from at least three independent biological replicates. (**C**) Western blots using BAG3 or actin antibodies from cells exposed to either control or heat shock (HS) and either DMSO or 100 nM PB, as indicated.

We previously established that inhibition of SF3B1 by different concentrations of pladienolide B (PB) causes distinct effects on the heat shock response (HSR) in HeLa cells [40]. The HeLa cell line was derived from cervical epithelial cells from a patient with adenocarcinoma. To examine the effects of PB in noncancerous cells, we chose the HEK293T cell line, which was derived from human embryonic kidney cells. Cell viability of HEK293T was measured using a Trypan Blue exclusion assay after exposure to varying doses of PB for 16 h (Figure 1A) [42]. We found that HEK293T cells displayed no loss of viability even when exposed to 100× the commonly used dose of 100 nM PB. Therefore, we concluded that the SF3B1 inhibitor pladienolide B (PB) is not cytotoxic to HEK293T cells.

To determine if the lack of cytotoxicity was due to overall resistance to PB, other hallmarks of SF3B1 inhibition were examined. First, we tested whether PB affected the splicing of a known SF3B1-dependent gene in HEK293T cells. As inhibition of SF3B1 in HeLa cells causes alternative splicing of the RBM5 transcript (skipping of exon 6), appearance of the alternatively spliced isoform of RBM5 (RBM5-AS) in HEK293T cells was quantitated using RT-qPCR [13,40]. A 1.3-fold increase in alternative splicing was observed relative to no drug at 5 nM PB and an 18.4-fold increase was found at 100 nM PB (Figure 1B). Therefore, HEK293T cells display alternative splicing changes upon the addition of PB in a dose-dependent manner. Second, we previously reported that inhibition of SF3B1 in HeLa cells inhibits the heat shock response (HSR) [40]. To determine if the HSR was similarly affected in HEK293T cells, expression of a known heat shock inducible protein, BAG3, was detected using Western blot analysis in the presence of PB and/or heat stress [43]. BAG3 protein expression is decreased in the presence of PB and is no longer inducible upon heat shock (Figure 1C). Therefore, the HSR is also regulated by SF3B1 in HEK293T cells. As HEK293T cells are sensitive to PB but are resistant to its cytotoxic effects, we reasoned that the HEK293T cell line would be well-suited for transcriptomic analyses for the discovery of SF3B1-dependent transcripts in a non-cancer-derived cell line.

### 2.2. Transcriptomes of Cells Exposed to PB Reveal Massive Changes to Transcript Architecture

To identify other genes and pathways that are differentially sensitive to SF3B1 inhibition, we performed RNA sequencing (RNA-seq) on HEK293T cells subjected to low (5 nM) and high (100 nM) concentrations of PB for 16 h. As our previous work showed that SF3B1 was sensitive to heat stress, we also included cells exposed to a standard 1-h heat shock at 42 °C to determine if transcriptome changes upon heat shock overlap with SF3B1 targets. RNA-seq was conducted on the Illumina HiSeq platform with paired-end 150-bp reads from polyA selected RNA. Since SF3B1 is known to be involved in splice site selection, the RNA-seq was performed at greater sequencing depth than typical for differential gene expression analysis (between 85 and 159 million reads), with each sample generating between 12 and 24 G of clean bases. Each condition was analyzed with biologically independent triplicates. The Pearson correlation coefficients between replicates ranged from 0.997 to 0.970. The Q20 was greater than 96% for all samples, indicating that the sequencing was of high quality.

**Figure 2:**
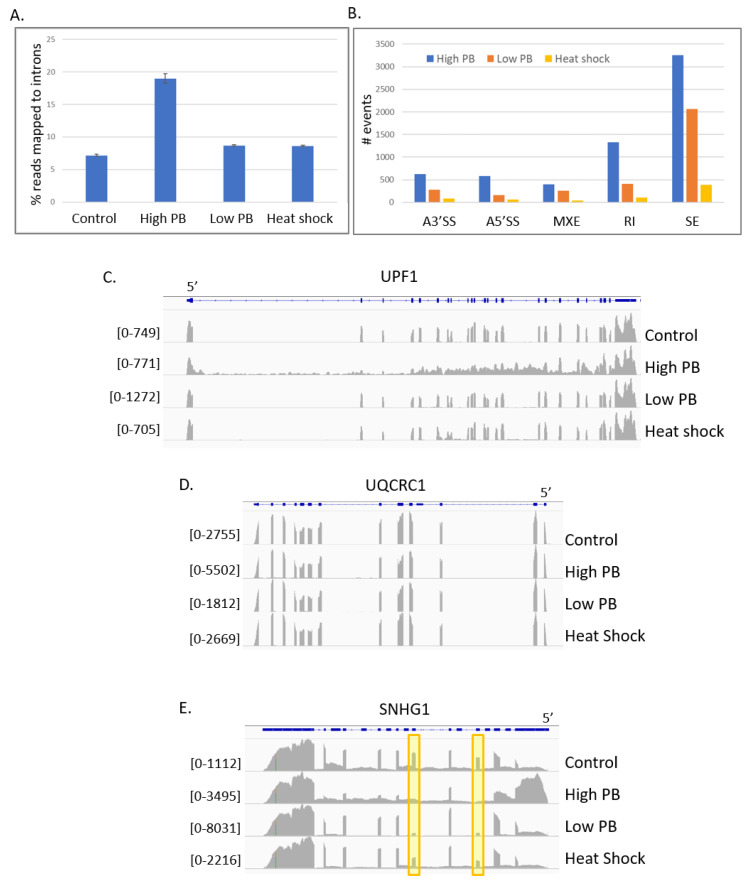
PB induces splicing changes to some genes but does not inhibit all splicing. RNA seq data from control, high PB (100 nM), low PB (5 nM) and heat shock samples. (**A**) Percentage of RNA-seq reads that mapped to intronic sequences. (**B**) Summary of mixture of isoforms (MISO) analysis showing alternative splicing changes. Each bar indicates the number of events detected compared to control cells with a percent spliced isoform (PSI) factor > 0.2 and a Bayes factor > 10. A3′SS = alternative 3′ splice site. A5′SS = alternative 5′ splice site. MXE = mutually exclusive exons. RI = retained intron. SE = skipped exon. (**C**) Integrative Genomics Viewer (IGV) genome view of RNA-seq reads to the UPF1 gene indicating widespread splicing defects in the high PB condition. (**D**) IGV genome view of RNA-seq reads mapping to the UQRCR1 gene showing unchanged splicing patterns in all conditions. (**E**) IGV genome view of RNA-seq reads mapping to the SNHG1 gene showing that different exons within a single gene can be sensitive to skipping in low PB vs. high PB. The highlighted exons are skipped in low PB while neighboring exons are correctly recognized. Bracketed numbers indicate data range for specified track.

Analysis of the transcriptome revealed that exposure to high PB concentration massively restructured transcript architecture, causing the percentage of reads mapping to intron sequences to increase from 7.2% to 19% of mapped reads (Figure 2A). These findings are consistent with the established role of SF3B1 in splicing. Exposure of cells to the low PB concentration also remodeled the transcriptome, but to a lesser degree, with the percentage of reads mapping to introns increasing to 8.7%. This change is on par with the change in intron reads occurring during heat stress at 8.6%. In contrast, reads mapping to intergenic regions remained stable in all conditions, ranging from 2.0% to 2.1% in control conditions, 2.1% to 2.2% in heat stress and the low drug condition and 2.4% to 2.6% in the high drug condition.

Changes in mRNA splicing patterns were analyzed using mixture of isoforms (MISO) analysis for five categories: alternative 5′ splice sites, alternative 3′ splice sites, mutually exclusive exons, retained introns and skipped exons [44]. To identify changes in splice patterns between conditions, results were filtered to include only events with a change in percent spliced isoform (PSI) factor greater than 0.2 and a Bayes factor threshold >10 relative to control samples (see Supplementary Materials File for complete list. The Bayes factor represents the likelihood that the isoform is differentially expressed. The summary of the MISO analysis shows that, as expected, high PB exposure induced a large number of alternative splicing events (Figure 2B). As a comparison, heat stress did not substantially increase alternative splicing events. Visualization of the MISO output for four alternative splicing events induced by the drug is shown as sashimi plots (Supplemental Figure S1). 

For many genes, high PB concentration caused an overall breakdown in accurate splicing, an example of which is shown with a genome view of reads mapped to the UPF1 locus with the IGV (Figure 2C). High PB (second track) displays a large change in transcript architecture with abundant intronic reads and variable exon usage throughout the locus. However, the disruption in splicing for UPF1 was largely restricted to the high PB condition as low PB (third track) displayed little change in splicing pattern compared to the control sample (top track). 

Although massive splicing changes are detected in the high PB condition, there is not a global defect in splicing as loci are visible that have no change in splicing pattern. For example, ubiquinol-cytochrome c reductase core protein 1 (UQCRC1), which also harbors a snoRNA, exhibits robust splicing along with a 1.9-fold increase in expression in high PB vs. control (Figure 2D).

The low PB transcriptome analysis allows the discovery of the specific introns which are the most sensitive to SF3B1. For example, SNHG1 shows differential exon expression at multiple exons in high PB but only two exons in low PB (Figure 2E). Notably, the same two exons affected in low PB are also affected during heat shock. Other examples of genes with differential splicing patterns between low and high drug conditions include proline and serine rich coiled-coil 1 (PSRC1) and ZNFX1 antisense RNA1 (ZFAS1) (snoRNA host gene) (Supplemental Figure S2). 

**Figure 3:**
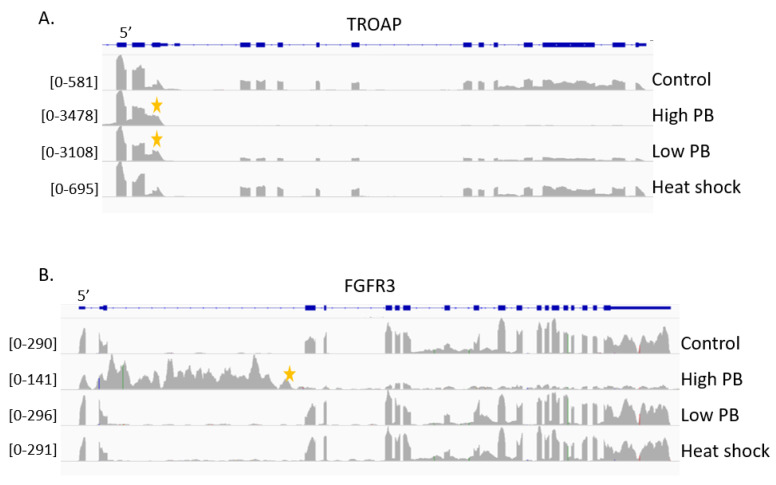
Early transcription termination is induced by PB. IGV genome view of RNA-seq mapped reads from control, high PB (100 nM), low PB (5 nM) and heat shock samples for (**A**) trophinin-associated protein (TROAP) and (**B**) fibroblast growth factor receptor 3 (FGFR3), showing early termination events, indicated with yellow stars. Bracketed numbers indicate data range for specified track.

Notably, a subset of transcripts displays patterns of particular interest as the mapped reads do not cover the 3′ end of the gene. These shortened transcripts imply early transcription termination events in a manner that is coupled to SF3B1 activity. These early termination events are visible at both low and high drug concentrations. Examples of early termination events include trophinin-associated protein (TROAP) and fibroblast growth factor receptor 3 (FGFR3) (Figure 3). TROAP has been associated with breast cancer proliferation and is an unfavorable prognostic marker for liver cancer (https://www.proteinatlas.org/ENSG00000135451-TROAP [45]). Early termination is visible in FGFR3 at high PB, whereas early termination is visible at both high PB and low PB for TROAP. The early termination signal for TROAP at low PB highlights that the early termination signal is not exclusive to high PB exposure and that a connection between SF3B1 and transcription termination exists.

### 2.3. PB induced Defect in NMD

**Figure 4:**
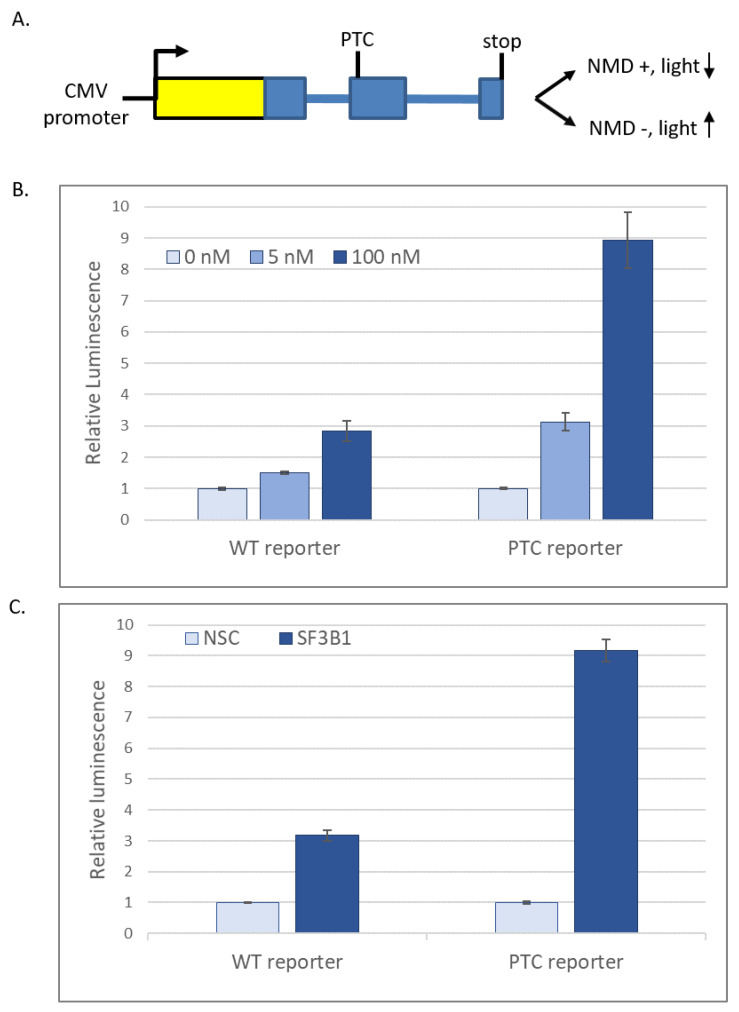
SF3B1 activity is required for nonsense-mediated decay (NMD). (**A**) Schematic of the NMD reporter system. Yellow = Firefly luciferase. Blue = human beta-globin gene. Boxes indicate exons and lines indicate introns. PTC indicates position of an introduced premature stop codon. Increased luciferase corresponds to decreased NMD activity. Ratio of luminescence from (**B**) 100 or 5 nM pladienolide B- vs. no-drug control- or (**C**) SF3B1 siRNA- vs. non-silencing (NSC) siRNA-treated cells containing the beta-globin-luciferase NMD reporter with either a premature termination codon (PTC reporter) or wild-type beta globin sequence (WT reporter).

We identified massively disrupted splicing for UPF1 at high PB (Figure 2C). UPF1 is an RNA-dependent helicase that is required for nonsense-mediated decay (NMD). In fact, multiple nonsense-mediated decay factors, including UPF2, UPF3b, SMG5, SMG7 and DHX34, show some degree of altered transcript architecture at high PB. A breakdown of the NMD machinery would lead to an accumulation of nonsense-bearing transcripts, which could cause the large, transcriptome-wide increases in intronic signal detected during high PB exposure (Figure 2A). To test this hypothesis, the status of the NMD pathway was quantitated using an existing reporter system [46]. This NMD reporter contains firefly luciferase in-frame with a human beta-globin gene construct containing two introns (Figure 4A). A premature termination codon (PTC) created within exon 2 of the beta-globin gene creates a substrate for NMD. In the presence of active NMD, the reporter is destroyed. The amount of light produced is inversely proportional to the degree of NMD inhibition, so that greater levels of NMD inhibition result in greater quantities of light produced. A wild-type (WT) reporter which does not contain an engineered PTC was used to control for effects on gene expression such as splicing that are NMD-independent. 

A striking increase in luminescence in the PTC-containing reporter relative to the WT reporter indicates that NMD is compromised (Figure 4B). Significantly, a similar increase was observed upon siRNA knockdown of SF3B1, revealing that this effect is not due to a non-specific effect of PB (Figure 4C). Together, these data suggest that NMD is greatly decreased during severe inhibition of SF3B1 activity. 

### 2.4. PB Induced Changes to Gene Expression

Changes to splicing patterns can have profound effects on transcript stability and gene expression. In order to identify other targets of SF3B1 regulation and to complement our efforts described above, we performed a differential gene expression analysis upon exposure to PB using DESeq2 [47]. Differentially expressed genes were constrained to a false discovery rate (FDR) of <1% (*p*-value < 0.01 by Benjamimi–Hochberg adjustment). Analysis of 100 nM PB vs. control resulted in the identification of 15,269 differentially expressed genes, which was considered the high PB regulon (see Supplementary Materials for complete list). Examination of the transcriptome upon exposure to 5 nM PB revealed that 10,754 genes show differential gene expression when compared to control cells. We considered this list to be the genes sensitive to low levels of SF3B1 inhibition, which we termed the low PB regulon (see Supplementary Materials for complete list).

Analysis of the data revealed that not all changes in expression correlate with changes in splicing pattern. For example, the few non-intron-containing genes in humans also display sensitivity to SF3B1 activity, including insulin receptor substrate 4 (IRS4) and H1.0 linker histone (H1F0). Moreover, several genes show reduced expression at low drug level and induced expression at high drug level but with no significant changes to splicing pattern, including inorganic pyrophosphatase 2 (PPA2), isocitrate dehydrogenase 3 non-catalytic subunit gamma (IDH3G), pyruvate dehydrogenase E1 subunit alpha 1 (PDHA1), peptidase D (PEPD) and heterogenous nuclear ribonucleoprotein C (HNRNPC). Therefore, detection of expression changes in the absence of splicing changes, particularly in the low drug condition, indicates that SF3B1 can influence gene expression other than by influencing splicing patterns. 

To determine if particular cellular pathways are more likely to be affected by PB exposure, a Gene Ontology (GO) term analysis was performed. The list of high PB and low PB regulons described above was split into those genes with >2-fold increase or decrease in expression and analyzed separately. Notably, no overlap in GO categories was detected between conditions, indicating that the two drug conditions may affect different cell processes (Table 1). 

**Figure 5:**
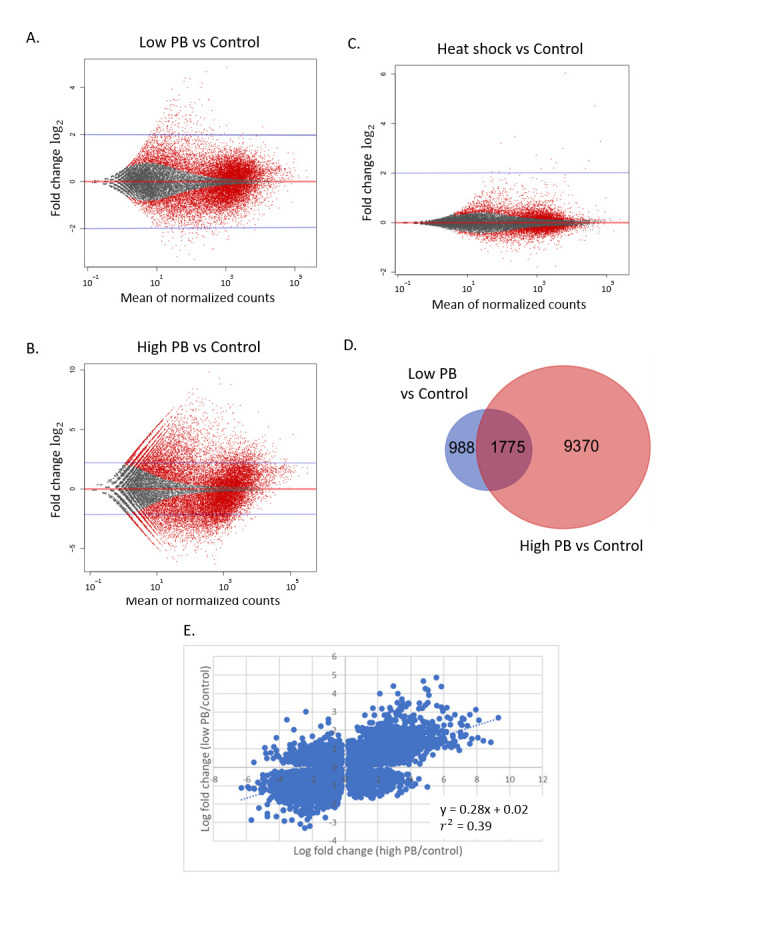
Dramatic changes in gene expression upon exposure to PB. M-A plots are shown depicting the mean normalized counts vs. log_2_ fold change relative to control for (**A**) low PB (5 nM), (**B**) high PB (100 nM) and (**C**) heat shock. Red points indicate genes with a padj. < 0.05. Grey points have padj. > 0.05. The red horizontal line marks no change between condition and control. The blue horizontal line indicates a 4-fold (log_2_ 2) change. (**D**) Venn diagram showing unique and overlapping targets at high PB vs. control and low PB vs. control. (**E**) Log fold change of the high PB vs. control ratio plotted against the low PB vs. control ratio.

The DESeq2 analysis also revealed that the genes with the greatest expression differences between low PB and control cells were typically expressed at low levels. Of the top 100 genes induced by low drug conditions, only one gene had an average normalized read count over 100 in control cells. To determine if this trend was true throughout the entire dataset, M-A plots were created with the mean of normalized counts on the *X*-axis and log_2_ fold changes on the *Y*-axis (Figure 5A). This analysis showed that a bolus of low expression genes with between 10 and 1000 mean counts was affected both positively and negatively by PB. Hundreds of genes in this range are induced by more than 2-fold, whereas few genes above 1000 counts are similarly affected.

While measurements of low-count genes are typically more error-prone and have higher *p*-values, it is important to note that a stringent 1% FDR cut-off was used here. Furthermore, in contrast to PB exposure, we found that the heat shock condition is not biased for genes with low expression (Figure 5C). This indicates that the impact of SF3B1 inhibition on low-count genes is not an artifact of the RNA collection or sequencing protocol and that the genes most affected by low PB were biased for low expression. Low transcript number does not equate to low cellular importance. Among the group of genes that are typically low in expression but induced in PB is amine oxidase copper-containing 3 (AOC3), induced by 11-fold in low PB and 14.6-fold in high PB, which is an unfavorable prognostic indication in renal, colorectal and urothelial cancers (https://www.proteinatlas.org/ENSG00000131471-AOC3, [45]).

Comparison of high drug (100 nM) vs. control shows that the overall effect on low-count genes is exacerbated, with 925 genes showing more than a 16-fold (log_2_ 4) change in expression over control cells (Figure 5B). In contrast to the low PB condition, high PB exposure induced a large number of significant expression changes throughout the transcriptome, in both low-count and high-count genes. Moreover, the magnitude of the expression changes at high PB was much larger than at low PB. Expression changes for low PB spanned from log_2_ −4.4 to log_2_ 3.3, while expression changes at high PB spanned from log_2_ −6.3 to log_2_ 9.9. We conclude that exposure to PB massively restructures the transcriptome and low-count genes are more sensitive to SF3B1 inhibition.

### 2.5. Sensitivity to Low PB Does not Predict Response to High PB

We expected that the low PB regulon would represent a subset of the high PB regulon that is most sensitive to PB activity. However, the GO term analysis above revealed no overlap between pathways affected in our conditions. To determine the extent of overlap between conditions, we first determined the proportion of genes that are significantly changed in expression in both the low PB and high PB conditions. We compared the list of genes with an expression change >2-fold and a padj. <0.01 in low PB vs. control against genes with the same cut-off values in high PB vs. control. Venn diagrams illustrate that differentially expressed genes only partially overlap (Figure 5D). In fact, more than a third (35.8%) of genes affected in the low PB condition are unique and do not display large expression changes in the high PB condition.

To determine if a more subtle correlation exists between the two conditions, we examined all statistically significant genes with no fold change cut-off by plotting the log fold change of high PB vs. control against the log fold change of low PB vs. control (Figure 5E). All genes that met the 1% FDR (padj. < 0.01) cut-off value in both datasets were included. The overall datasets show a weak positive correlation with an R^2^ value of 0.38, with many genes far from the diagonal. In fact, a significant number of transcripts are induced in low and repressed in high drug conditions, and vice versa (Figure 5E, upper left and bottom right quadrants, respectively). Therefore, the transcriptomic response to PB dosage is distinct.

To identify genes whose expression is most affected between high PB and low PB conditions, the normalized read counts from the high and low drug conditions were plotted directly against each other (Supplemental Figure S3). Using this methodology, we identified a particularly interesting set of genes that have a large decrease in expression in high drug conditions but show mild induction in low drug conditions, including FAM168B, SMC4, TRNC18, DEK and POLR2A. FAM168B is induced 2-fold in low drug and reduced 10.2-fold in high drug conditions. FAM168B is a favorable prognostic indicator in renal cancer and is known as a negative regulator of CDC42 and a positive regulator of CDC27 (https://www.proteinatlas.org/ENSG00000152102-FAM168B [45]). SMC4 is a component of the condensin complex and is an unfavorable prognostic marker in a number of cancers (https://www.proteinatlas.org/ENSG00000113810-SMC4 [45]). DEK is a proto-oncogene that has been linked to both DNA conformation and splice site selection and is known to be chromosomally translocated in a subset of acute myeloid leukemias [48]. POLR2A encodes the large catalytic subunit of RNA PolII and decreases 5.7-fold in expression when exposed to high drug levels. The HEK293T cells are still viable even though POLR2A is dramatically decreased. POLR2A displays the most significant decrease in expression of the RNA polymerase II component genes; however, it is not the only component affected. POLR2B displays a 1.3-fold decrease in expression, but POLR2E and POLR2I display a 4.7-fold and 8.2-fold increase in expression, respectively. On the other end of the spectrum, a number of genes increase in expression upon exposure to high drug levels (Supplemental Figure S3). The most extreme example is ZNF703 (highlighted in red). ZNF703 is induced 25-fold and is a transcriptional corepressor that is associated with breast cancer. Notably, multiple members of this short list play important roles in cancer. Interestingly, the splicing patterns for each gene listed here are either not affected or only mildly affected in low drug conditions but are greatly altered in high drug conditions. Considering the NMD pathway is compromised in high drug conditions, aberrantly spliced transcripts must be destroyed through an alternate RNA decay pathway, or the transcription of these genes may be targeted for regulation.

### 2.6. Low PB Enhances the Protein Folding Environment

We have previously shown that SF3B1 activity is sensitive to heat stress [40]. To determine if the transcripts sensitive to SF3B1 were also sensitive to heat stress, the PB-sensitive regulons and the heat stress regulon were compared. This analysis revealed that key components of the HSR but not the entire heat shock regulon display sensitivity to SF3B1 inhibition. Interestingly, the HSP40 family genes were more likely to be affected in low PB, high PB and heat stress conditions. The HSP40 family is a group of co-chaperones that regulate the ATPase activity of HSP70 chaperones. DNAJB1 was induced 26-fold in stress and 1.74- and 2.01-fold in high and low PB conditions, respectively. Other HSP40 family members were more strongly induced in low PB than in heat shock. DNAJC9 was induced 2.4-fold in low PB, 1.9-fold in high PB and 1.2-fold in heat stress conditions. DNAJB2 was induced more than 2-fold only in the low drug condition. Key chaperones are also negatively affected by SF3B1-inhibition; HSPA4L, DNAJC10 and DNAJC21 are reduced ~2.1-fold by at low drug concentrations. The decrease in expression is accompanied by visible changes in splicing pattern for HSPA4L and DNAJC21, but not for DNAJC10. In contrast, the HSP70 family members HSPA6 and HSPA1A were induced only in heat shock and not by either drug dose condition. 

**Figure 6:**
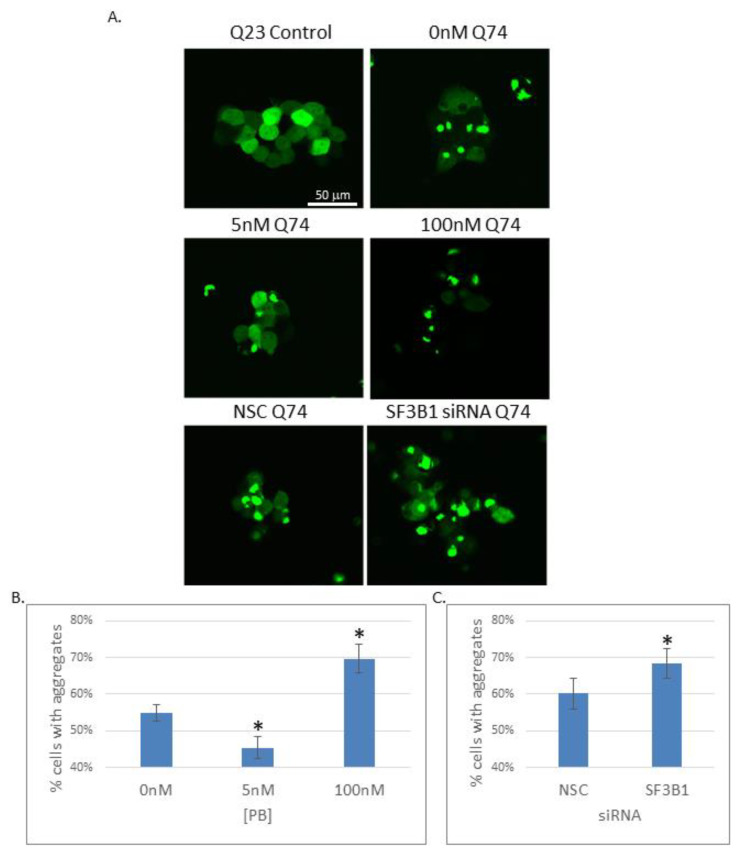
Protein-folding capacity is enhanced at low PB and inhibited at high PB conditions. (**A**) Fluorescent images showing cells containing a Q23-GFP control reporter or a Q74-GFP reporter exposed to low PB (5 nM), high PB (100 nM), non-silencing control (NSC) or SF3B1 siRNA treatments. (**B**) Quantitation of the percentage of cells with aggregates treated with PB. (**C**) Quantitation of cells treated with siRNA. Aggregates are scored when a cell contains at least one or more foci. Averages are shown representing data from three biological replicates per experiment and three independent experiments; error bars represent SEM. Asterix denotes *p* < 0.05.

Since the SF3B1 regulon includes key chaperone components of the proteostasis network, SF3B1 inhibition could alter the protein-folding balance in the cell. To test this hypothesis, the effects of PB were measured using a protein-folding sensor based on the huntingtin protein [49]. The length of the polyglutamine (polyQ) repeat region in the huntingtin protein correlates with a propensity for the protein to aggregate and is associated with Huntington’s disease. Protein misfolding and aggregation can be visualized in cells by fusing the repeat region to GFP. An aggregation-prone Q74-GFP reporter plasmid or an aggregation-resistant Q23-GFP control plasmid was transiently transfected with and without exposure to 5 and 100 nM PB (Figure 6A). A cell was scored as containing an aggregate if one or more foci were visible. Cells transfected with Q23-GFP showed diffuse localization with no foci formation in any conditions. In agreement with published results, we found that the Q74-GFP reporter aggregates in 55% of cells two days after transient transfection (Figure 6B). Strikingly, the number of cells containing aggregates increased to 69.7% upon 1 day exposure to a high drug concentration (100 nM PB), indicating a decrease in proteostasis. The increase in aggregation was also observed upon siRNA knockdown of SF3B1, indicating that off-target drug effects were not driving the increase in aggregation (Figure 6C). In contrast, the number of cells containing aggregates decreased to 45.3% with low drug concentration (5 nM PB), indicating that the general proteostasis environment appears more robust with low drug exposure. Together, these results reveal that the changes in chaperone expression observed upon PB exposure correspond with functional alterations in the protein-folding environment. Importantly, cellular proteostasis is differentially affected in low PB vs. high PB conditions, revealing a second physiological consequence of the expression changes observed in moderate vs. strong SF3B1 inhibition.

## 3. Discussion

To identify pathways that are differentially sensitive to SF3B1 activity, we have characterized the transcriptome of HEK293T cells exposed to low (5 nM) and high (100 nM) doses of the SF3B1 inhibitor pladienolide B. By comparing two levels of inhibition, we have identified the transcripts and introns that are the most sensitive to changes in SF3B1 activity. Unexpectedly, our data show that the genes affected by the low dose are not just a subset of those affected at the high dose, uncovering a new wealth of complexity. Significantly, these dose-dependent transcriptome changes reflect physiologically relevant effects on cell regulation, such as that nonsense-mediated decay is crippled during high drug inhibition, but low drug exposure actually improves the robustness of the protein-folding environment. Therefore, these data reveal that fine control of SF3B1 activity, shown here via the contrast of subtle vs. severe drug inhibition, has significant effects on the transcriptome resulting in dramatic physiological consequences. Particularly, since we have previously shown that SF3B1 is stress-sensitive, these subtle conditions are perhaps more reflective of physiologically relevant responses as opposed to conditions where SF3B1 activity is completely inhibited.

Remarkably, we observed a significant defect in NMD during exposure to high drug levels. NMD constitutes a major mRNA quality control pathway that degrades improperly processed, premature-codon-containing transcripts. The defect in the NMD pathway likely explains the large increase in intron signal observed in the RNA-seq analysis in the high drug level condition. In the low drug condition, at least seven proteins (UPF1, UPF2, UPF3b, DHX34, SEC13, SMG5 and SMG7) involved in NMD showed increased expression. However, the increase in expression of NMD factors in the low drug condition was not accompanied by a corresponding increase in NMD activity in our reporter assay. Therefore, this increase in expression is likely to be an indirect consequence of aberrant splicing which, in turn, causes an upregulation in mRNA quality control factors. 

NMD is known to undergo feedback regulation to meet cellular demand (reviewed in [50]). Interestingly, nonsense-mediated decay was identified here as a pathway with increased expression in high PB conditions (Table 1). This finding highlights that caution is necessary when interpreting transcriptomic data, particularly when known splicing and mRNA quality control measures have been affected. Increased mRNA expression is not equivalent to increased protein expression or activity. Here, the increase in expression was accompanied by a massive decrease in NMD activity, which was predicted by the change in splicing pattern and verified using independent reporter constructs (Figure 3). Nevertheless, GO analysis is useful to identify sensitive pathways, even if it cannot be used to determine whether that pathway is activated or repressed. 

The impact of SF3B1 on quality control in gene expression extends to protein homeostasis as well. A number of inducible and constitutive protein-folding chaperones show altered expression in the presence of PB which implies a change in the cellular proteostasis network. Considering that fewer Q74-GFP aggregates were observed in the low drug conditions illustrates a striking difference in the proteostasis environment. The increased aggregation of the Q74-GFP reporter during SF3B1 inhibition in high drug or SF3B1 knockdown conditions reveals a detrimental effect on proteostasis. The increased burden for the protein-folding machinery at high PB is likely due to the increased number of non-functional proteins caused by the defect in NMD. 

Notably, the range of effects observed at low PB is smaller than at high PB, but the number of genes that are affected remains substantial. The sequencing depth that was obtained and the number of replicates that were included enabled the application of stringent statistical criteria. This approach led to lower *p*-values and observations of effects for genes that were expressed at low levels which would have been missed by many typical RNA-seq experimental designs. Although low-expression genes are often ignored or filtered out of data analyses due to noise, thousands of genes displayed sensitivity to PB in a statistically significant manner here. Although many of these loci are annotated as pseudogenes and non-coding RNAs, recent work illustrating that many of these loci are translated and physiologically relevant suggests that it would be a mistake to ignore the consequences of changes to this section of the transcriptome [51]. Our data describe a large-scale remodeling of the transcriptome in response to inhibition of SF3B1 activity. 

This work underscores the importance of studying subtle changes to alternative splicing in order to comprehend the profound changes to expression and physiology that result. Determining which transcriptome changes are driven directly by regulation of SF3B1 and which changes are a downstream consequence of altered SF3B1 activity will require further investigation. In any case, it is clear that inhibition of the SF3B1 splicing factor has ramifications that resonate far beyond splicing and illuminate how a simple change in splice site can lead to complex changes at a systems level. 

We propose that the activity of SF3B1 is finely tuned in normal physiology. Common conditions such as heat stress alter SF3B1 activity, which alters splicing and gene expression. Although drug exposure is not natural, it provided an opportunity to identify the most sensitive SF3B1 substrates that are affected by a subtle decrease in SF3B1 activity. The physiological changes are meaningful, as illustrated by the dramatic difference in protein-folding environments reported here. Furthermore, by identifying substrates in a non-cancer-derived background, we can begin to understand the baseline role that SF3B1 plays in the cell. Understanding its native role can help to illuminate the mechanism of its dysregulation in cancer. Many of the transcripts we identified here play important roles in cancer. 

## 4. Materials and Methods 

### 4.1. Cell Culture 

HEK293T cells were a gift from the lab of Dr. Richard Morimoto and were grown in standard conditions (humid chamber, 5% CO_2_, 37 °C) in Dulbecco’s Modified Eagle Medium (DMEM) with GlutaMAX (Gibco/Thermo Fisher Scientific, Waltham, MA, USA) supplemented with 10% fetal bovine serum (FBS) and antibiotic-antimycotic (Gibco). Cells were maintained between 10% and 90% confluency and passaged less than 20 times from freezer stocks. Cell viability was measured using the CellTiter Fluor Cell Viability Assay (Promega, Madison, WI, USA) according to the manufacturer’s protocol.

### 4.2. RNA Harvest

HEK293T cells were grown to 50% confluency in 6-well plates and subjected to either 5 or 100 nM pladienolide B or DMSO only as a control. Pladienolide B was obtained from Santa Cruz Biotechnology (CAS 445493-23-2, Santa Cruz, CA, USA). Cells were grown under standard conditions with drug for 16 h. Cells were lysed directly in Trizol (Invitrogen/Thermo Fisher Scientific, Waltham, MA, USA) and the RNA was purified following established protocols [38]. The RNA samples were then split to allow subsequent analyses for quality control and RNA sequencing. All analyses were performed with a minimum of three biological replicates.

### 4.3. Transcript Quantitation

RNA samples were quantified on a nanodrop spectrophotometer. Samples were normalized for total RNA content and subjected to DNaseI treatment with the TURBO DNA-free kit (Invitrogen). The BioRad iScript kit was used for cDNA synthesis and the BioRad iTaq Universal SYBR supermix was used for amplification. All reactions were performed on a CFX96 Real-Time PCR detection system (BioRad, Hercules, CA, USA).

### 4.4. RNA Sequencing

RNA integrity was assessed using an Agilent 2100 bioanalyzer. PolyA RNA was purified from total RNA samples using poly-T oligo-attached magnetic beads. Library construction, RNA sequencing and preliminary data analysis were performed by Novogene. Paired-end 150-bp sequencing was performed using the Illumina HiSeq platform. Since we anticipated that alternative splicing events would be increased, the sequencing was performed at greater depth than typically used for differential gene expression analysis. The number of clean bases ranged from 12 to 23 G.

### 4.5. Transcriptome Analysis Pipeline

After quality control (QC) filtering, reads were aligned using TopHat2 [52] against the hg19 human genome build. Differential gene expression analysis was performed using DESeq2 [44]. The DESeq2 differential expression analysis was performed on each pair of conditions and plotted as indicated. Gene Ontology (GO) was performed with the reference list as a list of all genes that had at least 50 normalized read counts in any single experiment. Integrative Genomics Viewer (IGV) was used to visualize mapped reads. One representative replicate for each condition was used for visualization in the figures.

### 4.6. Trypan Blue Exclusion Assay

HEK293T cells were grown for 16 h either with DMSO or indicated concentrations of PB. Cells were trypsinized and an equal volume of 0.4% Trypan Blue (Thermo Fisher Scientific, Waltham, MA, USA) was added to the cell suspension. Cells were scored on a hemocytometer. Blue cells were scored as dead. As a positive control, 30 μg/mL digitonin was added to the cell suspension and incubated for 15 min.

### 4.7. Western Blots

HEK293T cells were grown in 6-well dishes. Cells were treated with 100 nM PB final concentration for ~16 h or with no drug as a control. The heat shock cells were incubated in a 42 °C water bath for 1 h, as described in [40], and allowed to recover at 37 °C for 6 h. Cells were harvested and immediately boiled for 10 min in sample buffer. Samples were separated on a standard Laemmli gel and transferred onto nitrocellulose. Blots were probed with anti-BAG3 (19, sc-136467, Santa Cruz Biotech) at 1:100 dilution or with the beta-actin antibody (C4, sc-47778, Santa Cruz Biotech) at 1:400 dilution. m-IgGк BP-HRP (sc-516102) was used as the secondary antibody at 1:1000 dilution. The Pierce ECL chemiluminescent Western blot detection system was used. Images were obtained on a ChemiDoc (BioRad). Biological replicates were performed on at least 3 independent days.

### 4.8. Nonsense-Mediated Decay Quantitation

NMD luciferase reporter constructs were obtained from Addgene. pKC4.06 (plasmid #112084) is the NMD sensor which contains the beta-globin gene fused to luciferase and a point mutation which creates a premature termination codon. pKC4.04 (plasmid #112085) is the corresponding control sensor, which is identical except for the absence of a premature termination codon. Plasmids were transfected into HEK293T cells grown on white 96-well plates using Lipofectamine 3000 (Thermo Fisher Scientific). A plasmid containing nanoluc (pNL1.1.PGK, Promega) served as a co-transfection normalization control. The Nano-Glo Dual-Luciferase Reporter Assay System (Promega) was used as directed. Light output was quantitated using a SpectraMax i3 microplate reader from Molecular Devices. Plasmids were a gift from James Inglese.

### 4.9. PolyQ Aggregation Assay

Huntingtin-derived reporter constructs were developed in the Rubinsztein lab and obtained from Addgene [48]. pEGFP-Q23 (plasmid #40261) contains partial exon 1 from HTT with 23 polyglutamine (Q) repeats and pEGFP-Q74 (plasmid #40262) contains 74 polyglutamine repeats. Cells were grown directly onto Nunc chambered coverglass (Fisher # 12-565-470) and transfected with Q23 or Q74 plasmid using Lipofectamine 3000 from Thermo Fisher Scientific. Cells were transfected on the first day, treated with or without 5 nM/100 nM PB on day 2 and then visualized on day 3 using a confocal microscope. For siRNA knockdowns, cells were co-transfected with plasmid and siRNA targeted to SF3B1 or using a non-silencing control. Silencer Select siRNAs were purchased from Invitrogen/Thermo Fisher Scientific (SF3B1 siRNA cat# 4392420, non-silencing control cat# 4390843). Cells were visualized 2 days after co-transfection.

### 4.10. Accession Numbers

Complete sequence reads are available at the Sequence Read Archive (SRA) under study number PRJNA685790 (https://www.ncbi.nlm.nih.gov/sra/PRJNA685790).

## Figures and Tables

**Table 1:** Gene Ontology (GO) pathways affected by PB.

	Increased Expression		Reduced Expression	
	Pathway	*p*-Value	Pathway	*p*-Value
High PB	Nonsense-mediated decay	9.66 × 10^−15^	Monocarboxylic acid transport	5.89 × 10^−4^
	SRP-dep cotrln protein targeting	7.28 × 10^−15^	Multicellular organismal signaling	3.71 × 10^−4^
	Cytoplasmic translation	5.74 × 10^−9^	Action potential	3.13 × 10^−4^
	Ribosomal large subunit assembly	3.98 × 10^−4^		
Low PB	Regulation of transcription	2.9 × 10^−12^	Cellular amino acid catabolic process	3.91 × 10^−5^
	Embryonic skeletal morphogenesis	3.92 × 10^−6^	Export across plasma membrane	3.52 × 10^−4^
	Nuclear division	1.08 × 10^−4^	Selective autophagy	3.4 × 10^−4^
			Neg regulation of axonogenesis	1.26 × 10^−4^

## Supplementary Materials

Supplementary materials can be found at https://www.mdpi.com/1422-0067/21/24/9641/s1.

## Abbreviations

PB	Pladienolide B
HSR	Heat Shock Response
DMEM	Dulbecco’s Modified Eagle Medium
FBS	Fetal bovine serum
FDR	False Discovery Rate
NMD	Nonsense-mediated decay
UTR	Untranslated region
A3′SS	Alternative 3′ splice site
A5′SS	Alternative 5′ splice site
MXE	Mutually exclusive exons
RI	Retained intron
SE	Skipped exon
snRNP	Small nuclear ribonucleoprotein
