# Supplementary material for: Titration of SF3B1 Activity Reveals Distinct Effects on the Transcriptome and Cell Physiology
Source: Int J Mol Sci. 2020 Dec 17;21(24):9641. doi: 10.3390/ijms21249641 (PMC7766730; doi:10.3390/ijms21249641)
Supplement: Supplementary file 1 [file ijms-21-09641-s001.zip › ijms-1025365 SI_KG edits.pdf]

# Titration of SF3B1 Activity Reveals Distinct Effects on the Transcriptome and Cell Physiology

**Karen S. Kim Guisbert *, Isiah Mossiah and Eric Guisbert**

Department of Biomedical and Chemical Engineering and Sciences, Florida Institute of Technology, Melbourne, FL, 32937 USA; imossiah2016@my.fit.edu (I.M.); eguisbert@fit.edu (E.G.)
* Correspondence: kkimguisbert@fit.edu

Received: 11 December 2020; Accepted: 14 December 2020; Published: date

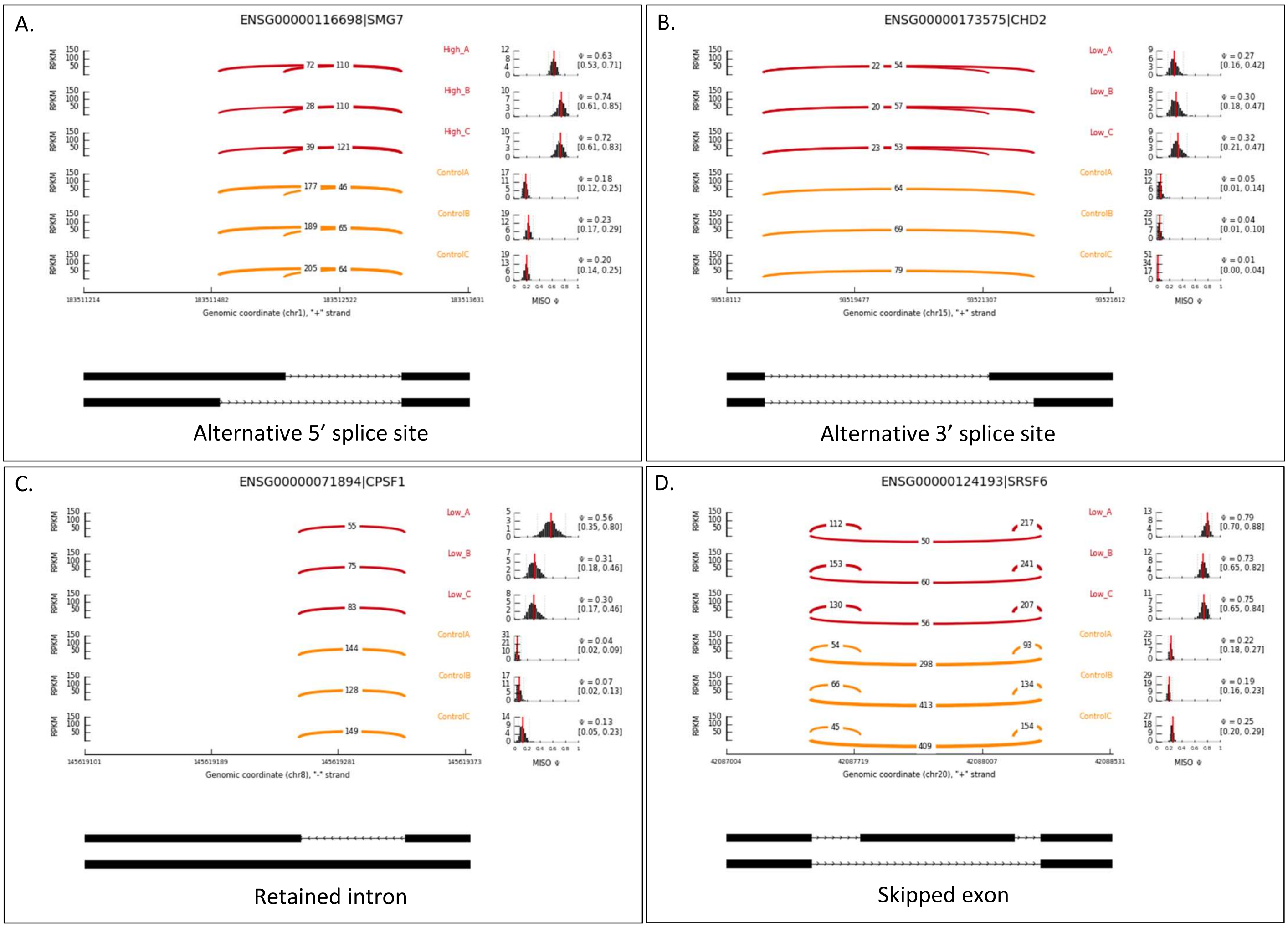


**Supplementary Figure 1.** Alternative splicing changes are induced in both low and high PB. Sashimi plots from MISO analysis graphing alternative isoforms generated in low or high PB compared with control for select genes. The number represents the number of reads spanning the junction from each biological replicate. The smaller chart on the right is the MISO PSI (percent spliced isoform) chart. (**A**) Alternative 5′ splice site in SMG7 transcripts in high drug vs control conditions. (**B**) Alternative 3′ splice site in CHD2 transcripts in low drug vs control conditions. (**C**) Retained intron in CPSF1 transcripts in low drug vs control conditions. (**D**) Skipped exon in SRSF6 transcripts in low drug vs control conditions.

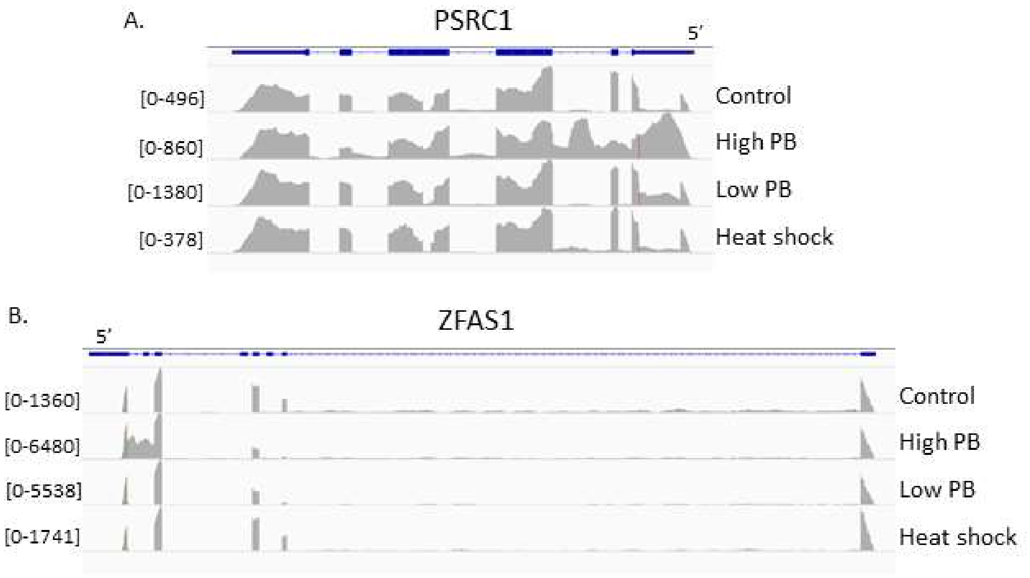


**Supplementary Figure 2.** Differential splicing pattern between conditions. IGV genome view of RNA-seq mapped reads from control, high PB (100nM), low PB (5nM) and heat shock samples for two genes showing differential effects on splicing. Differential splicing of transcripts from the (**A**) *PSRC1* locus and the (**B**) *ZFAS1* locus. Bracketed numbers indicate data range for specified track.

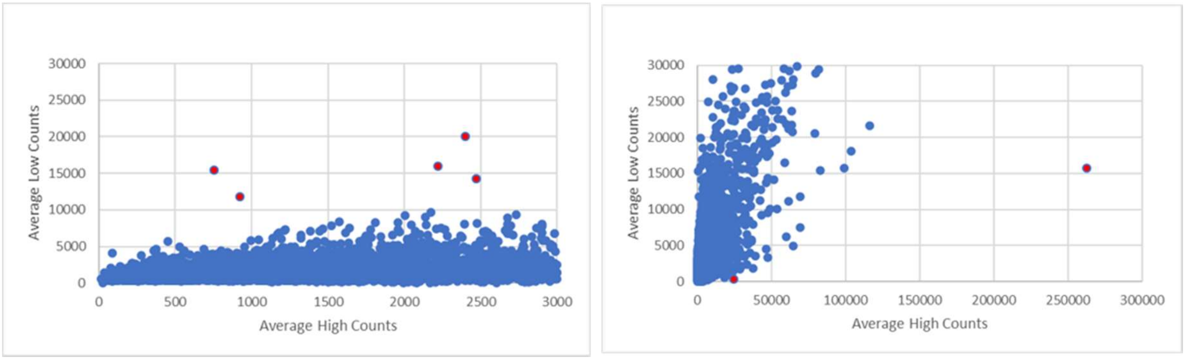


**Supplementary Figure 3.** Low PB and High PB can differentially affect gene expression. Scatter plots of mean counts in high PB vs mean counts in low PB to identify the most dramatic outliers (colored in red). Left panel: axes were adjusted to identify genes with many read counts in low PB but few read counts in high PB. Right panel: axes were adjusted to identify genes with many read counts in high PB but few read counts in low PB.
